# Weight loss might be an early clinical feature of undiagnosed human immunodeficiency virus infection in Taiwan

**DOI:** 10.1051/bmdcn/2018080319

**Published:** 2018-08-24

**Authors:** Shih-Wei Lai, Cheng-Li Lin, Kuan-Fu Liao

**Affiliations:** 1 College of Medicine, China Medical University Taichung 404 Taiwan; 2 Department of Family Medicine, China Medical University Hospital Taichung 404 Taiwan; 3 Management Office for Health Data, China Medical University Hospital Taichung 404 Taiwan; 4 College of Medicine, Tzu Chi University Hualien 970 Taiwan; 5 Division of Hepatogastroenterology, Department of Internal Medicine, Taichung Tzu Chi General Hospital Taichung 427 Taiwan

**Keywords:** Cohort study, Human immunodeficiency virus, Male, Taiwan National Health Insurance Program, Weight loss

## Abstract

Background/Objective: Little research is available on the relationship between weight loss and human immunodeficiency virus (HIV) infection in Taiwan. We hope to evaluate whether weight loss could be an early clinical feature of undiagnosed HIV infection in Taiwan.

Methods: We conducted a retrospective population-based cohort study using the database of the Taiwan National Health Insurance (NHI) Program. There were 4748 male subjects aged 1-84 with newly diagnosed weight loss as the weight loss group from 1998-2012 and 18982 age-matched male subjects without weight loss as the non-weight loss group. The incidence of HIV infection at the end of 2013 was measured in both groups. The multivariable Cox proportional hazards regression model was used to measure the hazard ratio (HR) and 95% confidence interval (CI) for HIV risk associated with weight loss.

Results: The overall incidence of HIV infection was 3.79-fold higher in the weight loss group than in the non-weight loss group (6.83 *vs.* 1.80 per 10000 person-years, 95% CI 3.41, 4.21). The incidence was the highest during the first 6 months of follow-up in the weight loss group (39.0 per 10000 person-years). After adjusting for confounding factors, the adjusted HR of HIV infection was 3.63 (95% CI 1.77, 7.44) for the weight loss group, compared with the non-weight loss group.

Conclusion: Weight loss might be an early clinical feature of undiagnosed HIV infection in Taiwan. Male patients with weight loss who have risk factors for HIV infection should be recommended to regularly be tested for HIV infection.

## 1. Introduction

Weight loss is clinically defined as a loss of 5% or more of one’s original body weight over 6 months.[1, 2] Weight loss is an important clinical problem with potential morbidity and mortality due to its correlation with underlying organic diseases or psychosocial diseases. The etiologies of weight loss are extensive, but a growing body of evidence indicates that the most frequent causes of weight loss may include malignancy, chronic inflammatory diseases, chronic infectious diseases, psychiatric diseases, gastrointestinal diseases, hyperthyroidism, diabetes mellitus, and human immunodeficiency virus (HIV) infection.[3–5]

The first HIV patient was detected in Taiwan in 1984. At the end of 2017, the total number of HIV cases among Taiwan’s people was 35935 (excluding foreigners), with males being overwhelmingly dominant (94.41%).[6] Previous studies indicate that many clinical features, including oral and peri-oral lesions, seborrheic dermatitis, genital warts, genital herpes, Pneumocystis carinii pneumonia, herpes zoster, and immune thrombocytopenic purpura [7–12] may herald the existence of an undiagnosed HIV infection.

To date, no formal systematic research focused on the relationship between weight loss and HIV infection has been conducted in Taiwan. If weight loss is an early clinical feature of an undiagnosed HIV infection, weight-loss patients who have risk factors for HIV infection should be recommended to be tested for an undiagnosed HIV infection. Therefore, we conducted a population-based cohort study using the database of the TaiwanNational Health Insurance Program to explore whether there is a relationship between weight loss and an undiagnosed HIV infection in Taiwan. Because the overwhelming majority of HIV cases in Taiwan have been male (94.41%) [6], we think that the outcome number of HIV infections among female weight-loss subjects would almost certainly be low. Thus, we only selected male subjects for our detailed analysis.

## 2. Methods

### 2.1. Study design

This was a retrospective population-based cohort study using the database of the Taiwan National Health Insurance Program. The program was implemented in March 1995, covering about 99.6% of the 23 million people living in the independent country of Taiwan [13–17]. The details of the program can be found in previous studies [18–23].

### 2.2. Study population

We identified male subjects aged 1-84 with newly diagnosed weight loss as the weight loss group from the period of 1998 to 2012, based on the International Classification of Diseases 9th Revision (ICD-9 code 783.21). The index date was defined as the date of subjects being diagnosed with weight loss. For each subject with weight loss, approximately 4 randomly selected male subjects without a diagnosis of weight loss were assigned to be part of the non-weight loss group. The weight loss group and the non-weight loss group were matched by age (every 5-year interval) and the year of index date. Subjects with HIV infection (ICD-9 codes 795.71, V08, 042, and 079.53) at the baseline in both groups were excluded from the study.

### 2.3. Comorbidities studied

Comorbidities in the study were included as follows: cancer (ICD-9 codes 140-208), diabetes mellitus (ICD-9 code 250), drug dependence (ICD-9 code 304), thyrotoxicosis (ICD-9 code 242), and venereal diseases (ICD-9 codes 090-099), which were all adapted from previous studies [9, 10, 24–38].

### 2.4. Main outcome

All study subjects were followed until they were newly diagnosed with HIV infection or until the end of 2013.

### 2.5. Statistical analysis

The distributions of age and comorbidities were compared between the weight loss group and the non-weight loss group by using a *Chi*-square test for categorized variables and the *t*-test for continuous variables. The incidence of HIV infection was measured as the event number of HIV infection identified during the follow-up period, divided by the total follow-up person-years for each group. Initially, all variables were included in the univariable Cox proportional hazards regression model. Variables found to be statistically significant in a univariable model were further examined in a multivariable Cox proportional hazards regression model to measure the hazard ratio (HR) and 95% confidence interval (CI) for the risk of HIV infection associated with weight loss and relevant comorbidities. The statistical significance level was set at a two-sided probability value of < 0.05. All analyses were performed by SAS software version 9.2 (SAS Institute Inc., Cary, North Carolina, USA).

## 3. Results

### 3.1. Baseline characteristics of the study population

Table 1 reveals the baseline characteristics of the study population between the weight loss group and the non-weight loss group. There were 4748 male subjects in the weight loss group and 18982 male subjects in the non-weight loss group, with a similar distribution of age. The mean ages (standard deviation) were 54.7(18.5) years for the weight loss group and 54.4 (18.6) for the non-weight loss group, without statistical significance (*t*-test, *P* = 0.23). The proportions of cancer, diabetes mellitus, drug dependence, thyrotoxicosis, and venereal diseases were equally distributed in both groups (*Chi*-square test, *P* > 0.05).

**Table 1:** Baseline characteristics of male subjects with and without weight loss.

	Non-weight loss N = 18982		Weight loss N = 4748		
Characteristic	n	(%)	n	(%)	*P* value*
Age group (years)					0.99
< 20	686	(3.6)	168	(3.5)	
20-39	3719	(19.6)	928	(19.6)	
40-64	8488	(44.7)	2128	(44.8)	
65-84	6089	(32.1)	1524	(32.1)	
Age (years), mean ± standard deviation†	54.4±18.6		54.7±18.5		0.23
Baseline comorbidities					
Cancer	684	(3.60)	177	(3.73)	0.68
Diabetes mellitus	1813	(9.55)	455	(9.58)	0.95
Drug dependence	62	(0.33)	16	(0.34)	0.91
Thyrotoxicosis	692	(3.65)	174	(3.66)	0.95
Venereal diseases	237	(1.25)	61	(1.28)	0.84

Data are presented as the number of subjects in each group with percentages given in parentheses.

* *Chi*-square test, and

† *t*-test comparing subjects with and without weight loss.

### 3.2. Incidence of HIV infection of the study population

Table 2 reveals that the overall incidence of HIV infection was 3.79-fold higher in the weight loss group than that in the nonweight loss group (6.83 *vs*. 1.80 per 10000 person-years, 95% CI 3.41, 4.21). The incidences of HIV infection, as stratified by age and follow-up period, were all higher in the weight loss group than those in the non-weight loss group. The weight loss group aged 20-39 had a higher incidence of HIV infection (13.3 per 10000 person-years). The analysis stratified by follow-up period revealed that the incidence seemed to be the highest during the first 6 months of follow-up in the weight loss group (39.0 per 10000 person-years).

**Table 2:** Incidence of human immunodeficiency virus infection estimated by age and follow-up period between male subjects with and without weight loss.

	Non-weight loss				Weight loss					
Variable	N	Event	Person-years	Incidence‡	N	Event	Person-years	Incidence‡	IRR*	(95% CI)
All	18982	16	88694	1.80	4748	14	20502	6.83	3.79	(3.41, 4.21)
Age group (years)										
< 20	686	1	3570	2.80	168	0	875	0.00	-	-
20-39	3719	10	18572	5.38	928	6	4526	13.3	2.46	(1.95, 3.10)
40-64	8488	3	40383	0.74	2128	6	9554	6.28	8.45	(7.07, 10.1)
65-84	6089	2	26168	0.76	1524	2	5547	3.61	4.72	(3.94, 5.65)
Follow-up period (months)										
< 6	18982	2	9440	2.12	4748	9	2310	39.0	18.6	(16.1, 21.4)
≥ 6	18783	14	79254	1.77	4512	5	18191	2.75	1.59	(1.41, 1.80)

‡ Incidence rate: per 10000 person-years.

* IRR (incidence rate ratio): weight loss *vs.* non-weight loss. (95% confidence interval)

Among the weight loss group, 14 subjects were diagnosed with HIV infection. Of these 14 subjects, 64.3% (9/14) were detected to have HIV infection during the first 6 months of follow-up (2 subjects aged 21-30, 3 subjects aged 31-40, 3 subjects aged 41-50, and 1 subject aged 51-60). In addition, 35.7% (5/14) were detected to have HIV infection after 6 months (1 subject aged 2130, 1 subject aged 41-50, 1 subject aged 51-60, and 2 subjects aged 71-80). Totally, 85.7% (12/14) were younger subjects (aged 21-60).

### 3.3. HIV infection associated with weight loss and comorbidities

After adjusted for confounding factors, the multivariable Cox proportional hazards regression model revealed that the adjusted HR of HIV infection was 3.63 (95% CI 1.77, 7.44) for the weight loss group, compared with the non-weight loss group. In addition, drug dependence (adjusted HR 13.0, 95% CI 1.75, 96.4), and venereal diseases (adjusted HR 22.9, 95% CI 9.23, 56.9) were other factors significantly related to HIV infection (Table 3).

**Table 3:** Hazard ratio and 95% confidence interval of human immunodeficiency virus infection associated with weight loss and comorbidities in male subjects.

	Crude		Adjusted†	
Variable	HR	(95%CI)	HR	(95%CI)
Age (per one year)	0.96	(0.94, 0.98)	0.96	(0.94, 0.98)
Weight loss (yes *vs.* no)	3.69	(1.80, 7.55)	3.63	(1.77, 7.44)
Comorbidities (yes *vs.* no)				
Cancer	1.10	(0.15, 8.11)	-	-
Diabetes mellitus	0.34	(0.05, 2.49)	-	-
Drug dependence	12.1	(1.65, 89.3)	13.0	(1.75, 96.4)
Thyrotoxicosis	0.90	(0.12, 6.59)	-	-
Venereal diseases	19.5	(7.97, 47.9)	22.9	(9.23, 56.9)

† Variables found to be statistically significant in a univariable model were further examined in a multivariable model. Adjusted for age, drug dependence, and venereal diseases.

## 4. Discussion

In this retrospective cohort study, as mentioned in the Methods Section, patients with weight loss were selected before they had a confirmed diagnosis of HIV infection. We noticed that the overall incidence of HIV infection was 3.79-fold higher in the weight loss group than that in the non-weight loss group. The incidence was the highest during the first 6 months of follow-up in the weight loss group (Table 2). We noticed that the risk of HIV infection in the weight loss group still persisted over time, even after 6 months (Table 2). We think this kind of risk is related to the latent state of HIV infection. This point suggests that if male patients with weight loss have risk factors for HIV infection, physicians should always keep in mind the possibility of HIV infection even if initially HIV infection is not detected. Thus, these high-risk patients should be recommended to be regularly tested for undiagnosed HIV infection.

After adjusting for confounding factors, patients with weight loss were associated with 3.63-fold increased hazard of HIV infection (Table 3). Previous studies have documented that weight loss could be regarded as one of the constitutional symptoms associated with HIV/AIDS-related wasting syndrome [39, 40], To the contrary, weight loss also can be one of the acute-phase features of undiagnosed HIV infection before serologic detection of HIV [41–43], and this is compatible with our finding that weight loss can be regarded as an early clinical feature of an undiagnosed HIV infection.

In further analysis of this study, we noticed that the number needed to be screened for HIV infection was 126.3 among patients aged 21 to 30, 183 among patients aged 31-40, 186 among patients aged 41-50, and 484.5 among patients aged 51-60. In fact, not all patients will have a risk for HIV infection. Similarly, not all weight-loss patients need to test for HIV infection. Therefore, only those who have risk behaviors associated with HIV exposure should be recommended to be tested for HIV infection. Whenever weight-loss patients go for consultation, physicians should ask for a detailed history, including intravenous drug use, needle sharing, history of sexual contact, and history of unsafesex practices, to decide who should be recommended to be tested for HIV infection, especially when dealing with younger male patients. Thus, the number needed to be screened can be reduced. During the process of risk assessment, physicians can educate the patients about risk reduction, such as safe sex and safe drug use.

Some limitations of this study should be mentioned. First, due to the natural limitation of the claim data, some traditional risk factors for HIV infection, such as intravenous drug use and history of sexual contact, could not be assessed using the registry data. Therefore, drug dependence was included instead of injecting drug use and venereal diseases were included instead of history of sexual contact. This limitation has been mentioned in previous studies [9, 10]. Second, due to the same limitation, whether the weight loss was involuntary or voluntary could not be determined. Moreover, patients who looked for consultation about weight loss are those worried about their health status. We think that their weight loss should thus be considered to be involuntary. Third, due to the same limitation, we could not determine how much weight loss was found in each patient. We could only use the ICD-9 code of weight loss (ICD-9 code 783.21) instead. The validity of the diagnostic code of weight loss could not be assessed using the registry data. Fourth, due to the same limitation, other symptoms associated with undiagnosed HIV infection were not recorded in the database. We could not determine whether weight loss or other symptoms came first.

The symptoms of HIV infection can differ from person-to-person, and some patients may not get any symptoms at all for many years. In different disease progressions, patients with HIV infection would have different symptoms. This study cannot tell the readers the stages of HIV infection in the study population. Fifth, the event number of HIV infection was too small to believe firmly in the statistical power of the study (14 events in the weight loss group and 16 events in the non-weight loss). A further study with a much larger event number of HIV infection is required to confirm the relationship between weight loss and HIV infection. Sixth, surveillance bias should be taken into account. Patients with weight loss might frequently perform routine testing to search for the cause of their weight loss. As a result, these patients were more likely to be detected for HIV infection. Seventh, the hazard of HIV infection for patients with drug dependence or patients with venereal diseases seemed to be higher than weight loss itself (Table 3), but we just hope to emphasize that weight loss is associated with an increased hazard of HIV infection. Eighth, some comorbidities, such as chronic inflammatory diseases, chronic infectious diseases, psychiatric diseases, and gastrointestinal diseases, could be associated with weight loss. Due to no specific ICD-9 codes for these comorbidities, we were unable to include them for analysis. If there are specific ICD-9 codes for these comorbidities in the future, they can be included for detailed analysis.

Some strengths of this study deserve mentioning. Despite not being a novel issue, this is the first epidemiological study based on a well-organized national database to evaluate the relationship between weight loss and HIV infection in Taiwan. The study design is relatively rational. The method is well documented. The results are very straight-forward. The discussion and literature review are markedly extensive. This study provides updated knowledge on weight loss and HIV infection in Taiwan.

We conclude that weight loss is associated with a 3.63-fold increased hazard of HIV infection. Weight loss might be an early clinical feature of an undiagnosed HIV infection in Taiwan. We emphasize again that male patients with weight loss who have risk factors for HIV infection should be recommended to be regularly tested for undiagnosed HIV infection, especially younger male patients.

## Specific author contributions

Shih-Wei Lai contributed to the conception of the article, initiated the draft of the article, and revised the article.

Cheng-Li Lin conducted the data analysis and revised the article.

Kuan-Fu Liao participated in the data interpretation and revised the article.

## Conflict of interest statement

The authors wish to disclose no conflicts of interest.

## Ethical statement

The insurance reimbursement claims data used in this study were available for public access. Patient identification numbers were scrambled to ensure confidentiality. Patient informed consent was not required. This study was approved by the Research Ethics Committee of China Medical University and Hospital in Taiwan (CMUH-104-REC2-115).
